# UHRF1 regulates global DNA hypomethylation and is associated with poor prognosis in esophageal squamous cell carcinoma

**DOI:** 10.18632/oncotarget.11067

**Published:** 2016-08-05

**Authors:** Kenichi Nakamura, Yoshifumi Baba, Keisuke Kosumi, Kazuto Harada, Hironobu Shigaki, Keisuke Miyake, Yuki Kiyozumi, Mayuko Ohuchi, Junji Kurashige, Takatsugu Ishimoto, Masaaki Iwatsuki, Yasuo Sakamoto, Naoya Yoshida, Masayuki Watanabe, Mitsuyoshi Nakao, Hideo Baba

**Affiliations:** ^1^ Department of Gastroenterological Surgery, Graduate School of Medical Science, Kumamoto University, Kumamoto, Japan; ^2^ Department of Gastroenterological Surgery, Cancer Institute Hospital, Japanese Foundation for Cancer Research, Tokyo, Japan; ^3^ Department of Medical Cell Biology, Institute of Molecular Embryology and Genetics, Kumamoto University, Kumamoto, Japan

**Keywords:** LINE-1, methylation, esophageal cancer, prognosis, UHRF1

## Abstract

**Background:**

Global DNA hypomethylation contributes to oncogenesis through various mechanisms. The level of long interspersed nucleotide element-1 (LINE- 1) methylation is considered a surrogate marker of global DNA methylation, and is attracting interest as a good predictor of cancer prognosis. However, the mechanism how LINE-1 (global DNA) methylation is controlled in cancer cells remains to be fully elucidated. Ubiquitin-like with PHD and RING finger domain 1 (UHRF1) plays a crucial role in DNA methylation. UHRF1 is overexpressed in many cancers, and UHRF1 overexpression may be a mechanism underlying DNA hypomethylation in cancer cells. Nonetheless, the relationship between UHRF1, LINE-1 methylation level, and clinical outcome in esophageal squamous cell carcinoma (ESCC) remains unclear.

**Results:**

In ESCC cell lines, vector-mediated UHRF1 overexpression caused global DNA (LINE-1) hypomethylation and, conversely, *UHRF1* knockdown using siRNA increased the global DNA methylation level. In ESCC tissues, UHRF1 expression was significantly associated with LINE-1 methylation levels. Furthermore, UHRF1 overexpression correlated with poor prognosis in our cohort of 160 ESCC patients.

**Materials and Methods:**

The relationships between UHRF1 expression and LINE-1 methylation level (i.e., global DNA methylation level) were investigated using ESCC tissues and cell lines. In addition, we examined the correlation between UHRF1 expression, LINE-1 methylation, and clinical outcome in patients with ESCC.

**Conclusions:**

Our results suggest that UHRF1 is a key epigenetic regulator of DNA methylation and might be a potential target for cancer treatment.

## INTRODUCTION

Esophageal squamous cell carcinoma (ESCC) is one of the most malignant tumors [1]. Despite remarkable progress in the advance of multidisciplinary treatments combining surgery, chemotherapy and/or radiotherapy, the outcome of ESCC patients remains unfavorable even after complete resection [2–4]. To develop novel strategies for treatment of ESCC, especially tumors that are molecularly targeted [5], it is extremely crucial to increase our understanding of the molecular basis of this disease. In particular, including alterations of DNA methylation, epigenetic changes are reversible and could be potential targets for cancer treatment and chemoprevention [6–8].

Alterations in DNA methylation correlated with human cancers include site-specific CpG island promoter hypermethylation and global DNA hypomethylation [9]. Global DNA hypomethylation contributes to oncogenesis through various mechanisms, including genomic instability [10–12]. Because long interspersed nucleotide element-1 (LINE-1) represents a considerable part of human genome (approximately 17%), LINE-1 methylation levels have been considered as a surrogate marker of global DNA methylation [13]. We have previously described that LINE-1 hypomethylation robustly correlates with poorer outcome in some cancers, including esophageal, gastric, and liver cancers [14–18], implying that LINE- 1 hypomethylation might be an attracting biomarker of predicting patient outcome. In addition, we found that LINE-1 hypomethylation in ESCC might contribute to the acquirement of malignant tumor behavior through genomic gains of oncogenes such as *CDK6* [19]. However, the mechanism by which LINE-1 (and hence global DNA) methylation is controlled in ESCC cells remains to be fully explored.

Ubiquitin-like with PHD and RING finger domain 1 (UHRF1) plays a crucial role in DNA methylation by recognizing hemimethylated DNA during DNA replication and recruiting DNA methyltransferase 1 (DNMT1) to preserve DNA methylation pattern in daughter cells [20–25]. UHRF1 has been shown to be highly expressed in many cancers, and UHRF1 overexpression is mechanism of DNA hypomethylation in tumor cells. Mudbhary et al. demonstrated that plasmid-mediated UHRF1 overexpression in zebrafish delocalized and destabilized Dnmt1 and caused DNA hypomethylation. Additionally, they found that UHRF1 overexpression correlated with poor patient outcome in human hepatocellular carcinoma [26].

In this study, we investigated the relationship between UHRF1 expression and LINE-1 methylation level (i.e., global DNA methylation level) using ESCC samples and ESCC cell lines. Furthermore, we analyzed the correlation between UHRF1 expression, LINE-1 methylation, and clinical outcome in ESCC.

## RESULTS

### Relationship between UHRF1 expression and LINE-1 methylation levels in ESCC tissues

**Figure 1:**
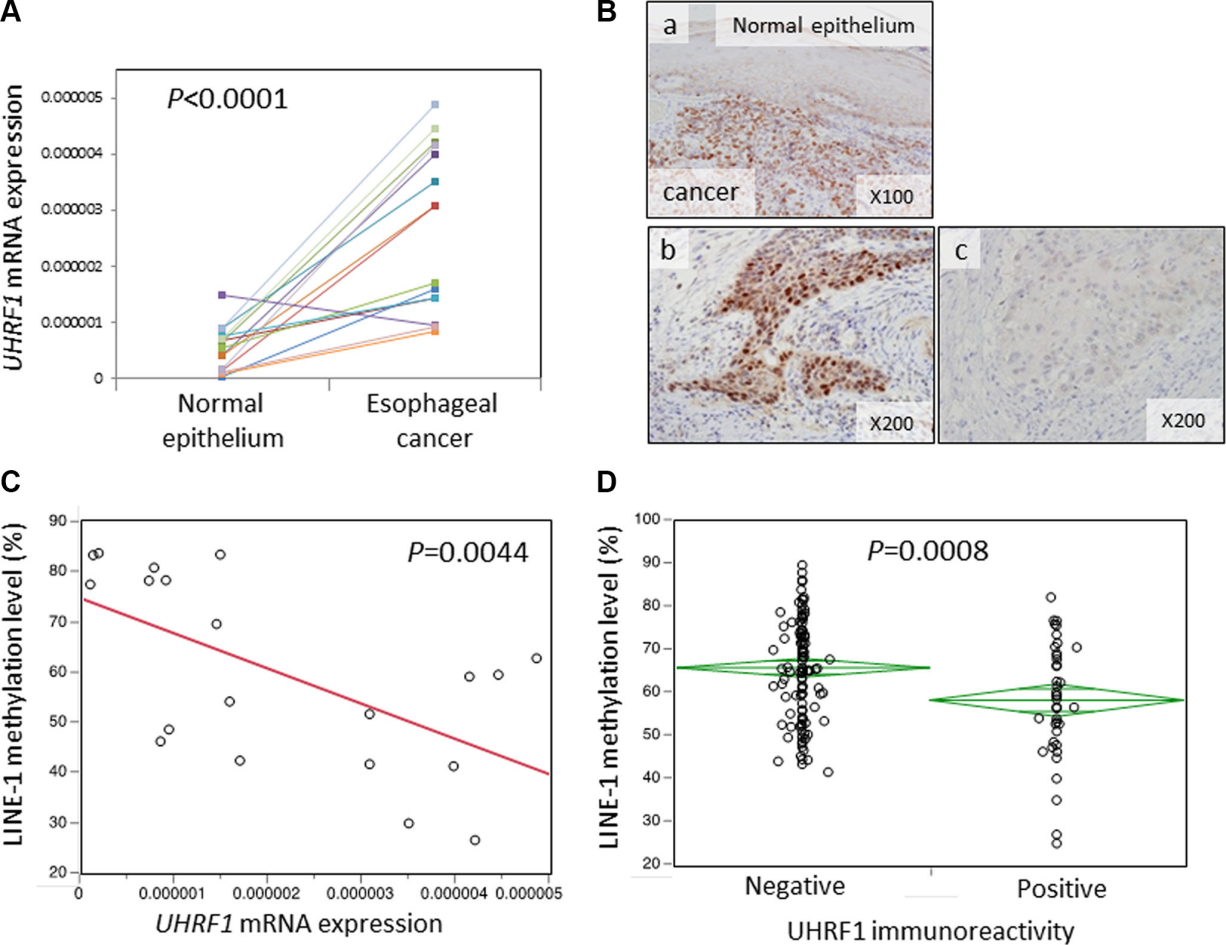
Relationship between UHRF1 expression and LINE-1 methylation levels in ESCC tissues (**A**) *UHRF1* mRNA expression levels in esophageal cancers and matched normal mucosa (*N* = 16). The cancer tissues showed significantly higher levels of expression than the matched normal mucosa (*P* < 0.0001 by paired *t*-test). (**B**) UHRF1 immunostaining of esophageal cancer and normal esophageal mucosa. (a) Cancerous lesions show positive staining whereas normal mucosa shows negative staining. (b) Positive expression of UHRF1 in nuclei of esophageal cancer cells. (c) Negative expression of UHRF1 in nuclei of esophageal cancer cells. (**C**) *UHRF1* mRNA expression levels were negatively associated with LINE-1 methylation levels (*P* = 0.0044). (**D**) UHRF1-positive tumors showed significantly lower levels of LINE-1 methylation than UHRF1-negative tumors (*P* = 0.00080 by paired *t*-test).

First, we measured *UHRF1* mRNA expression levels by qRT-PCR in 16 frozen esophageal cancer tissues and matched normal mucosa. *UHRF1* mRNA expression levels were significantly higher in cancer tissues than in normal mucosa (*P* < 0.0001, Figure 1A). Next, we carried out immunohistochemical analysis of UHRF1 protein expression in ESCCs. UHRF1 immunoreactivity was weak in normal esophageal mucosa. Among 160 ESCC tumors, 40 tumors (25%) showed positive staining of UHRF1 and 120 tumors (75%) showed negative staining (Figure 1B).

We next examined the relationship between UHRF1 expression and LINE-1 methylation levels. We measured LINE-1 methylation levels in 160 ESCC tissues and found that LINE-1 methylation inversely correlated with *UHRF1* mRNA expression (*P* = 0.0044, Figure 1C) and protein immunoreactivity (*P* = 0.008, Figure 1D). These findings support a relationship between UHRF1 expression and LINE-1 hypomethylation (i.e., global DNA hypomethylation) in ESCC tissues. *UHRF1* mRNA expression in 16 frozen esophageal cancer tissues did not correlate with UHRF1 protein expression in FFPE sections of the same cases by IHC. This was agreement with previous finding [27].

### Vector-mediated UHRF1 overexpression caused DNA hypomethylation in ESCC cell lines

**Figure 2:**
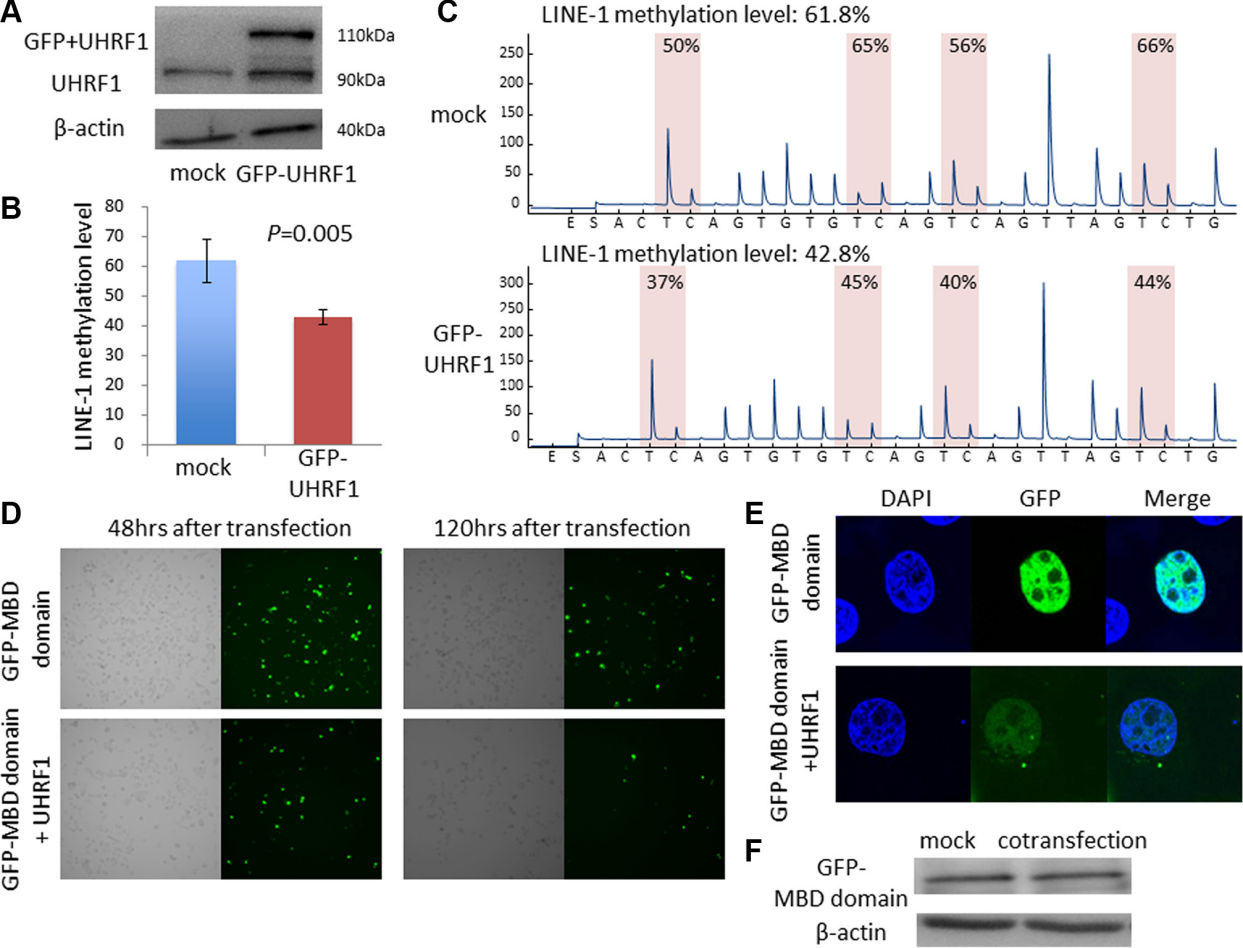
Vector-mediated UHRF1 overexpression caused DNA hypomethylation in ESCC cell lines (**A**) Western blot analysis of UHRF1 expression in KYSE30 cells treated with GFP-fused UHRF1 vector. (**B**) Vector-mediated UHRF1 overexpression caused LINE-1 hypomethylation (*P* = 0.005). (**C**) Pyrograms for LINE-1 methylation levels in KYSE30. (**D**, **E**) Changes in DNA methylation after co-transfection with UHRF1 vector and EGFP-MBD1 vector. (**F**) Expression of EGFP-MBD1 after co-transfection determined by western blot analysis.

To examine whether UHRF1 overexpression can influence LINE-1 methylation level (global DNA methylation level) in esophageal cancer cell lines, we transfected KYSE-30 cells, which exhibited low expression of UHRF1 and LINE-1 hypermethylation, with UHRF1 vector (Figure 2A) and analyzed LINE-1 methylation levels using a bisulfite PCR pyrosequencing assay. LINE-1 methylation levels of KYSE-30 cells transfected with UHRF1 plasmid were significantly decreased compared with those transfected with vector control (Figure 2B, 2C). We next cotransfected cells with UHRF1 plasmid and pEGFP-fused MBD1 (methyl-CpG binding domain 1) vector to confirm that overexpression of UHRF1 caused global DNA hypomethylation. The MBD1 is a component of methyl CpG binding protein1 and binds specifically methylated CpG sequences in the DNA; thus, pEGFP-fused MBD1 vector can be used to visualize global DNA methylation [28]. EGFP expression was decreased in the cancer cells that were cotransfected with EGFP-MBD1 and UHRF1 plasmid compared with cells transfected with EGFP-MBD1 alone. Decreased expression was shown overall and for individual cancer cells (Figure 2D, 2E). We confirmed that the expression level of MBD1 itself did not change after co-transfection (Figure 2F).

### Knockdown of *UHRF1* caused upregulation of global DNA methylation levels in ESCC cell lines

**Figure 3:**
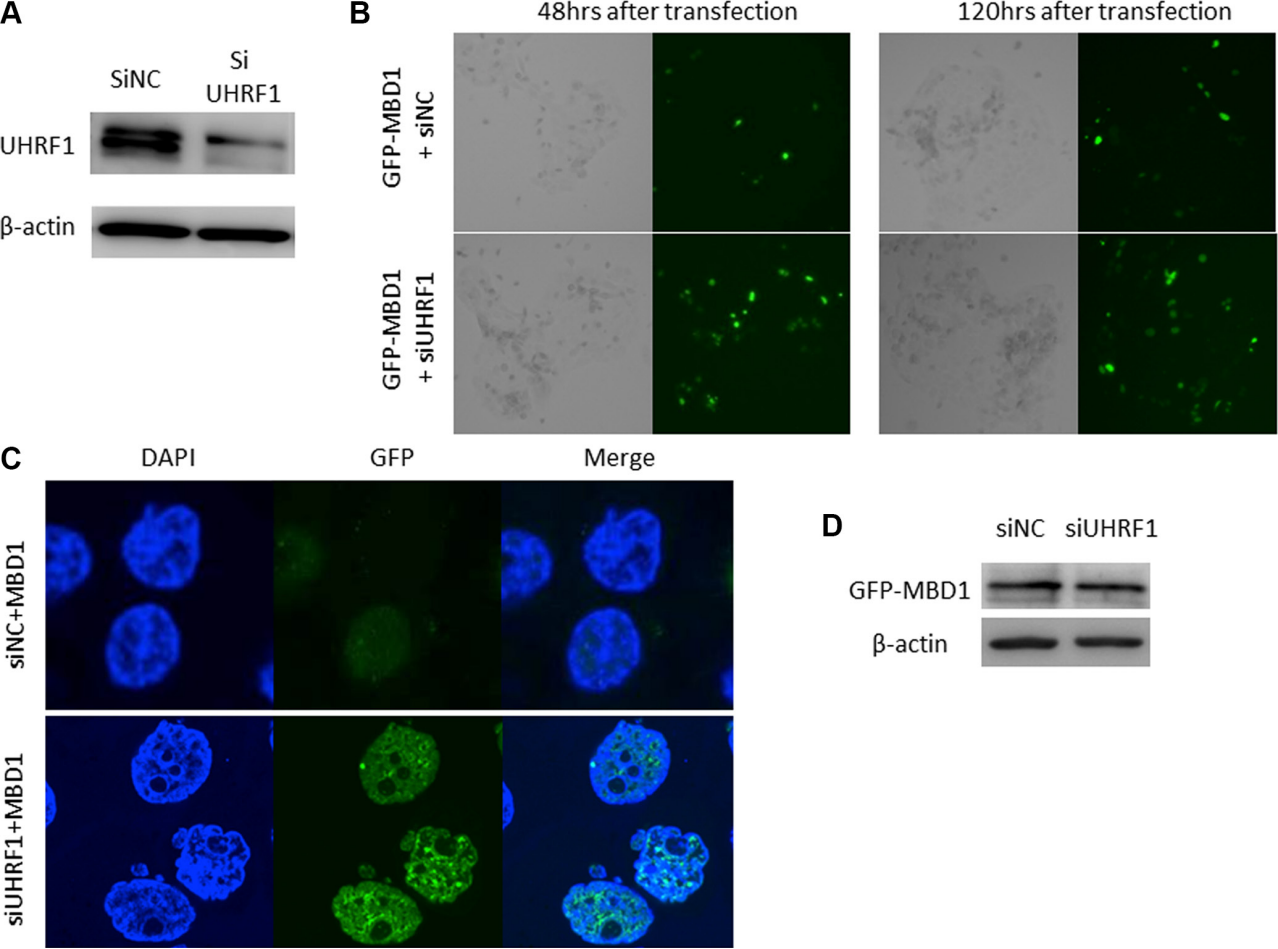
Knockdown of *UHRF1* with siRNA caused upregulation of LINE-1 methylation levels in ESCC cell lines (**A**) Western blot analysis of UHRF1 expression in cells transfected with siUHRF1. (**B**, **C**) Changes in DNA methylation after cotransfection with siUHRF1 and EGFP-MBD1 vector. (**D**) EGFP-MBD1 expression after cotransfection, as determined by western blotting.

Conversely, to investigate whether *UHRF1* knockdown increased global DNA methylation level we transfected TE-11 cells, which exhibited high expression of UHRF1 and LINE-1 hypomethylation, with siRNA specific for *UHRF1* (Figure 3A). EGFP expression increased in the cancer cells that were cotransfected with EGFP-MBD1 and siUHRF1, compared with cancer cells transfected with EGFP-MBD1 and the normal control siRNA (siNC) overall and in individual cancer cells (Figure 3B, 3C), whereas expression of MBD1 itself did not change (Figure 3D). These results suggested that UHRF1 regulates the global DNA methylation level.

### Changes of UHRF1 expression level in ESCC cell lines treated with 5AZA

To confirm that the decrease in DNA methylation level does not affect UHRF1 expression level, we treated 10 ESCC lines with 5AZA. Although LINE-1 methylation level decreased in all ESCC cell lines after 5AZA treatment, there were no significant changes in UHRF1 mRNA and protein expression (Supplementary Figure S1A, S1B). These findings suggested that the changes in DNA methylation level did not influence *UHRF1* expression level (Supplementary Figure S1C).

### UHRF1 expression and patient outcome

**Figure 4:**
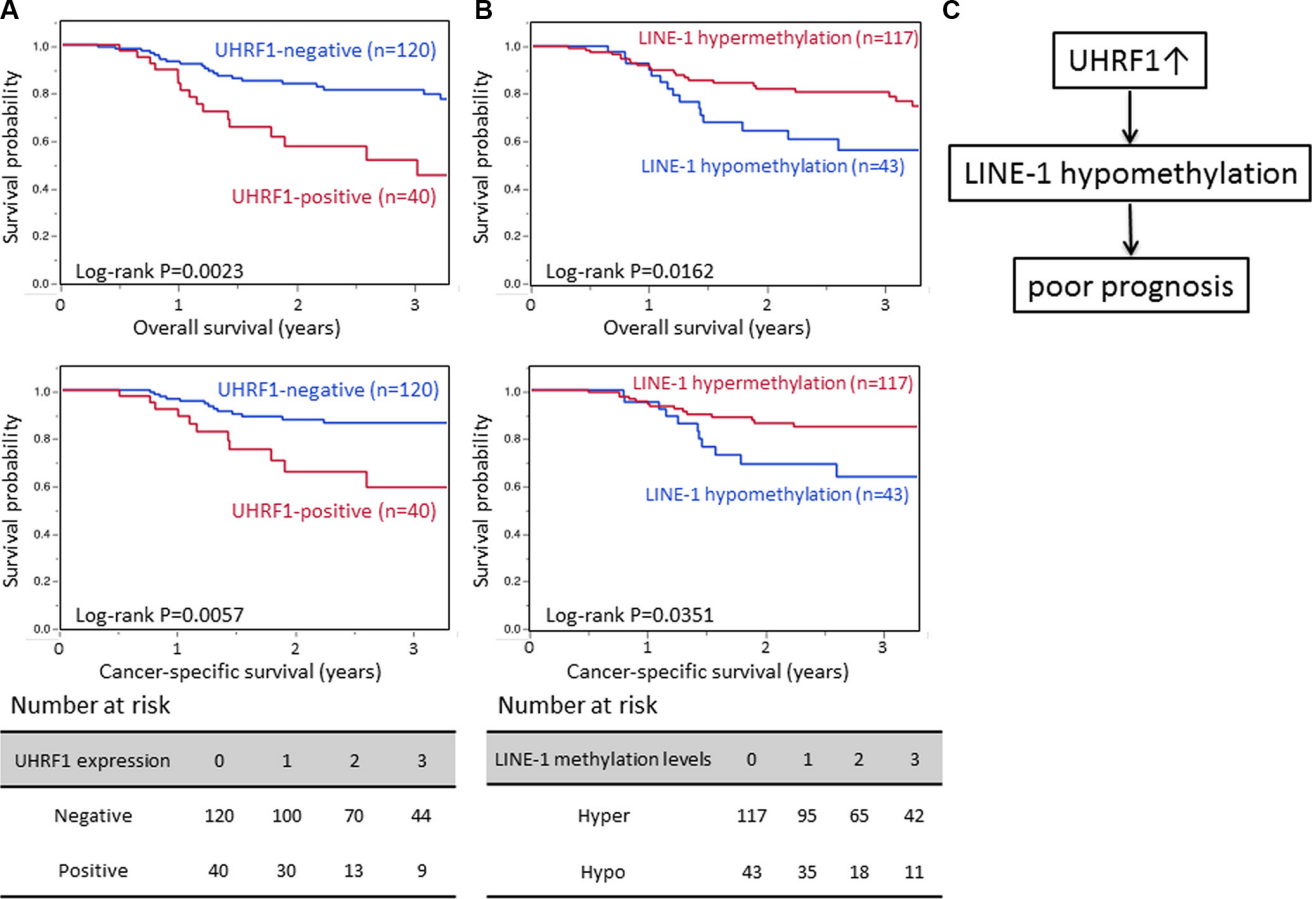
Survival analyses of UHRF1 expression and LINE-1 methylation level (**A**) Kaplan–Meier curves according to UHRF1 expression status. (**B**) Kaplan–Meier curves according to LINE-1 methylation status. (**C**) Possible mechanism by which UHRF1 confers a poor prognosis in ESCC.

Table 1 shows the relationship between UHRF1 expression levels and clinical and pathological characteristics. There was no relationship between UHRF1 expression and clinical and pathological characteristics including sex, age, tumor location, tumor size, T and N stage, and microscopic lymphovascular invasion. In the Kaplan–Meier analysis, the UHRF1-positive group (*n* = 40) exhibited significantly poorer overall survival (OS) and cancer-specific survival (CSS) than the UHRF1-negative group (*n* = 120) (3-year OS rate 51.4% vs. 81.0%, log-rank *P* = 0.0023; 3-year CSS rate 59.1% vs. 85.1%, log-rank *P* = 0.0057; Figure 4A).

**Table 1:** Relationship beween UHRF1 expression, clinical and pathological features

Clinical and pathological features	Total N	UHRF1 expression		*P* value
		Negative	Positive	
**All cases**	160	120	40	
**Mean age (years) ± SD**	65.9 ± 9.0	66.3 ± 9.4	65.0 ± 7.5	0.44
**Sex**				0.49
Male	139	103	36	
Female	21	17	4	
**Tumor location**				
Upper	97	69	28	0.17
Lower	63	51	12	
**Tumor size (mm, mean ± SD)**	41.2 ± 20.7	40.6 ± 19.9	42.9 ± 23.6	0.64
**T stage**				0.51
T1	84	66	18	
T2	21	16	5	
T3	54	37	17	
T4	1	1	0	
**N stage**				0.24
Negative	82	65	17	
Positive	78	55	23	
**Stage**				0.12
I	55	47	8	
II	57	38	19	
III	42	31	11	
IV	6	4	2	
**Lymphatic invasion**				0.37
Negative	113	87	26	
Positive	47	33	14	
**Venous invasion**				0.92
Negative	79	59	20	
Positive	81	61	20	

We next evaluated whether the influence of UHRF1 expression status on patient outcome was modified by LINE-1 methylation levels. According to our previous report [23], we defined LINE-1 hypomethylation as 55.5. In Kaplan–Meier analysis, the LINE-1 hypomethylation group (*n* = 43) exhibited significantly poorer OS and CSS than the LINE-1 hypermethylation group (*n* = 117) (3-year OS rate 56.1% vs. 78.8%, log-rank *P* = 0.0162; 3-year CSS rate 63.7% vs. 84.7%, log-rank *P* = 0.0351; Figure 4B). A similar tendency was seen in univariate Cox regression analysis, in which the UHRF1-positive group experienced significantly poorer OS and CSS (HR for OS = 2.61, 95% CI 1.35–4.92, *P* = 0.0050, HR for CSS = 2.72, 95% CI 1.21–5.91, *P* = 0.0161. In the LINE- 1 adjusted Cox model, the HR of UHRF1 was decreased to 2.15 (95% CI 1.09–4.15, *P* = 0.0277) for OS, and 2.52 (95% CI 1.20–5.51, *P* = 0.0265) for CSS (Table 2). This result shows a proportional reduction in the regression coefficient for UHRF1 expression due to the inclusion of LINE-1 hypomethylation in the Cox regression model (Figure 4C). In the multivariate analysis, UHRF1 was an independent prognostic factor (HR for OS = 2.42 95% CI 1.24–4.60, *P* = 0.0101, and HR for CSS = 2.55, 95% CI 1.13–5.59, *P* = 0.0248).

**Table 2:** Univariate and LINE-1 adjusted Cox regression analysis for OS and CSS

UHRF1 expression	Overall survival		Cancer-specific survival	
	Univariate HR (95% CI)	LINE1-adjusted HR (95% CI)	Univariate HR (95% CI)	LINE1-adjusted HR (95% CI)
negative	1 (reference)	1 (reference)	1 (reference)	1 (reference)
	**2.61**	**2.15**	**2.72**	**2.52**
positive	(1.35–4.92)	(1.09–4.15)	(1.21–5.91)	(1.20–5.51)
*P* value	0.0050	0.0277	0.0161	0.0265

## DISCUSSION

We have previously reported that LINE-1 hypomethylation (global DNA hypomethylation) is strongly associated with poorer patient outcome in some cancers, including esophageal, gastric, and liver cancers [14–18], implying that LINE-1 hypomethylation may be a useful prognostic biomarker in gastrointestinal cancers. However, the mechanism by which the LINE- 1 methylation level is regulated remains unclear. In the present work, we demonstrated that vector-mediated UHRF1 overexpression caused global DNA hypomethylation and, conversely, *UHRF1* knockdown using siRNA caused global DNA hypermethylation *in vitro*. Furthermore, we showed a proportional reduction in the regression coefficient for UHRF1 expression due to the inclusion of LINE-1 methylation in the Cox regression model. Collectively, our data indicate that UHRF1 may regulate global DNA methylation, and that UHRF1 overexpression contributes to an unfavorable prognosis in patients with ESCC via global DNA hypomethylation (Figure 4C).

Accumulating evidence suggests that UHRF1 is overexpressed in human cancers and plays a crucial role in malignant tumor behavior. UHRF1 is essential for cell growth and UHRF1 overexpression has been reported to promote cell proliferation whereas depleting UHRF1 leads to cell cycle arrest by inhibiting G1/S transition [29]. Moreover, cancer cells with UHRF1 overexpression present enhanced rates of growth and migration and morphologic features resembling epithelial mesenchymal transition (EMT) [30]. In addition, UHRF1 plays a pivotal role in the regulation of gene expression through epigenetic mechanisms including DNA methylation, histone methylation [31], histone deacetylation [32], and histone ubiquitination [33]. The mechanism by which UHRF1 overexpression causes global DNA hypomethylation has been proposed to involve DNMT1 delocalization and destabilization [25], ubiquitination and degradation [34], or redistribution and isolation of DNMT1 away from DNA [35]. Excess UHRF1 might isolate USP1, a DNMT deubiquitination enzyme, thus promoting DNMT1 ubiquitination and degradation. Our study also supports an epigenetic role of UHRF1 in esophageal cancer.

As LINE-1 constitutes a substantial portion (approximately 17%) of the human genome, the LINE- 1 methylation level is considered to be a useful marker of global DNA methylation [10]. We confirmed that vector-mediated UHRF1 overexpression caused LINE-1 hypomethylation. However, as the level of LINE-1 methylation is just a surrogate marker, we also demonstrated that vector-mediated UHRF1 overexpression caused global DNA hypomethylation using a pEGFP-fused MBD1 vector. MBD1 is a member of methyl CpG binding domain (MBD) family. The MBD domain of MBD1 performs the canonical function of recognizing the methyl group at the methylated DNA site through a hydrophobic patch that consists of five highly-conserved MBD amino acid residues. As MBD1 specifically binds methylated CpG sequences in the DNA, GFP localization should also be specific for methylated CpG sequences [28]. Because the EGFP-MBD1 fusion protein does not affect methylated DNA sequences, it is useful for monitoring genomic DNA methylation patterns [36].

UHRF1 overexpression [28, 29] has been reported to correlate with unfavorable prognosis in many cancers, including breast [37], lung [38, 39], colorectal [30, 40], prostate [41], bladder [42, 43], and gastric cancer [44]. Previous study has reported that UHRF1 overexpression correlated significantly with advanced T-stage, positive lymph node metastasis and poor differentiation in ESCC, however There was no relationship between UHRF1 expression and clinical and pathological characteristics in our study [45]. One mechanism by which UHRF1 overexpression causes unfavorable outcome is through phenotypic changes of the tumors. Inhibition of *UHRF1* using siRNA decreased cellular proliferation and migration in colorectal cancer cell lines [40]. Although we examined the influence of UHRF1 expression on phenotype using siRNA and overexpression vectors, these phenotypic changes did not occur in ESCC cell lines (data not shown). Another mechanism is through inactivation of various tumor suppressor genes. Excess UHRF1 was shown to be localized on the methylated promoters of these genes and suppressed expression through transcriptional repressors such as G9a and HDAC1 [38, 42]. However, few reports have analyzed the relationship between UHRF1 overexpression and global DNA methylation. In the current study, we demonstrated that UHRF1 overexpression contributed to poor prognosis in ESCC, possibly through LINE-1 hypomethylation. Furthermore, we examined whether the influence of UHRF1 overexpression on overall survival was modified by any of the clinical and pathological variables; however, none of the covariates exerted a significant effect in the survival analysis (overall survival, *P* for interaction > 0.09 in all tests, Supplementary Figure S2).

The mechanism how global DNA hypomethylation causes a poor prognosis remains unclear. Some studies have shown that global DNA hypomethylation correlated with chromosomal instability and mitotic catastrophe [9, 25, 26, 45, 46]. The hypomethylation of transposons causes an open chromatin conformation and promotes oncogene activation. For instance, a LINE-1 element that is inserted into the c-MET gene drives the transcription of c-MET, which is termed L1-MET [47]. Moreover, LINE-1 hypomethylated ESCC tumors exhibit highly frequent copy number gains at various loci [18]. Another study reported that genomic DNA hypomethylation was associated with inflammatory mediators and oxidative stress [48]. Further studies are needed to validate our findings and clarify the mechanism how LINE-1 hypomethylation affects tumor behavior.

In summary, vector-mediated UHRF1 overexpression caused LINE-1 (global DNA) hypomethylation and, conversely, *UHRF1* knockdown using siRNA increased DNA methylation. In addition, UHRF1 overexpression in ESCC correlated with poor prognosis. These results suggest that UHRF1 is a key epigenetic regulator of DNA methylation, and, moreover, might be an attractive target for cancer therapies. Future studies are necessary to confirm our findings and to investigate potential mechanisms how global DNA hypomethylation caused by UHRF1 overexpression affects tumor progression.

## MATERIALS AND METHODS

### Study subjects

A total of 168 patients were randomly selected from 276 patients with esophageal cancer who underwent surgical resection irrespective of preoperative treatment (chemotherapy, radiation therapy, or chemoradiotherapy) at Kumamoto University Hospital (Kumamoto, Japan) between February 2005 and November 2011. Eight patients were excluded because of inadequate tissue samples, therefore 160 patients were finally enrolled in this study. Among this cohort studies, there was no significant difference in clinical and pathological characteristics between this study group and the excluded group (data not shown). Total RNA was obtained from 16 frozen ESCC tumors and matched with macroscopically noncancerous mucosa from the same patient. To assess the prognostic impact of UHRF1 expression, we performed immunohistochemical staining of 160 ESCC samples. This current analysis represents a new analysis of UHRF1 on the existing esophageal cancer database that has been previously characterized for LINE-1 methylation and clinical outcome [15], which is analogous to novel studies using the well-described cell lines or animal models. The American Joint Committee on Cancer (AJCC) Staging Manual (7th edition) was used for tumor staging.

We defined disease-free survival as the time between the date of surgery and recurrence. Overall survival was defined as the time between the date of surgery date and the date of death. A follow-up study of the 160 patients revealed 49 recurrences and 43 deaths, including 25 esophageal cancer-specific deaths. The median time for censored patients was 2.7 years. We obtained written informed consent from all patients, and the protocol of this study was approved by the Institutional Review Board.

### Measurement of LINE-1 methylation by Pyrosequencing

Genomic DNA (gDNA) was collected from frozen esophageal cancer specimens with a QIAamp DNA Mini Kit (Qiagen, Valencia, CA). gDNA was converted with sodium bisulfite by an EpiTect Bisulfite kit (Qiagen). We performed polymerase chain reaction (PCR) and pyrosequencing for LINE-1 as previously described [15] with a PyroMark kit (Qiagen). A region of LINE-1 element (position 305 to 331, accession No. X58075) was amplified, including four CpG sites. Using a PyroMark Q24 System (Qiagen), pyrosequencing reactions were performed. Bisulfite-pyrosequencing consists of three steps: bisulfite conversion, PCR amplification, and pyrosequencing analysis. Unmethylated cytosine (C) and methylated cytosine (^m^C) are differentiated by bisulfite treatment followed by PCR. In the pyrosequencing step, the ratio C:^m^C at each CpG site is measured as the ratio of T:C (where T represents thymine). The C content relative to the C plus T content at each CpG site is expressed as a percentage. In this study, the average relative C content at the four CpG sites was considered as overall LINE-1 methylation level in the tumor.

### Immunohistochemical staining

After deparaffinizing tissue blocks, antigen epitope retrieval was performed in antigen retrieval solution using a streamer autoclave at 120°C for 15 minutes (pH 9, Histofine; Nichirei Biosciences Inc., Japan). In order to block endogenous peroxide enzyme, tissue sections were incubated for 30 minutes using Peroxidase-Blocking Solution (Dako, S2023). After apply of primary antibodies specific for UHRF1 (1:100 dilution; ab57083; Abcam, Cambridge, UK), the slides were incubated overnight at 4°C. Secondary antibody, anti-mouse EnVision^TM+^/HRP (Dako Japan Inc., Tokyo, Japan), was applied and hematoxylin counterstained. UHRF1 expression was assessed by a pathologist who was blinded to other data. We considered UHRF1 immunoreactivity as positive when the cancer cell nuclei were stained homogenously. We used a dual scoring system of staining extent and intensity for immunohistochemical analysis. All specimens stained for UHRF1 were interpreted independently by two pathologists (Y.B. and K.N.), unaware of other data. The concordance between the two observers was 0.90 (k = 0.72, *p* < 0.0001), indicating good agreement.

### Quantitative reverse transcription polymerase chain reaction

Extraction of total RNA, synthesis of cDNA, and quantitative reverse transcription PCR (qRT-PCR) were performed as previously reported [26, 27]. Primers for qRT-PCR were designed with the Universal Probe Library (Roche, Basel, Switzerland) following the manufacturer's recommendations. The primers for real-time PCR were as follows: *UHRF1*, Hs00273589_m1 (Taqman probe, Applied Biosystems, Foster City, CA), and *18S*, Hs99999901_S1 (Taqman probe, Applied Biosystems).

### Cell lines

ESCC cell lines of human (KYSE-30 and TE series) were acquired from the Japanese Collection of Research Bioresources Cell Bank, the Cell Resource Center for Biomedical Research, and the Riken BioResource Center Cell Bank. These cell lines were cultured in RPMI 1640 or DMEM added with 10% FBS in a 5% CO_2_ atmosphere at 37°C.

### UHRF1 suppression by silencer select small-interfering RNAs

We used two chemically synthesized UHRF1-specific small-interfering RNAs (siRNAs) (s26553 and s26554, Life Technologies). The negative control was silencer select RNAi negative control (Life Technologies). We transfected cells with 10 nM UHRF1-siRNAs or control siRNA using Lipofectamine RNAiMAX (Life Technologies). Cells were collected at 48 hours post-transfection and retained for assays.

### Plasmid construction

The cDNA clone encoding full-length human *UHRF1* was obtained using RNA of TE-11 cells as a template and the following gene-specific primers containing Nhe I and Xho I sites (underlined): forward primer (5′-TAGCTAGCCACCATGTGGAT CCAGGTTCGGACCATG-3′) and reverse primer (5′-CGC TCGAGCCGGCCATTGCCGTAG-3′). *E. coli* (DH5a) was transformed with the resultant plasmid followed by selection and culture of the positive clones and isolation of *UHRF1* cDNA. *UHRF1* cDNA was introduced into the pIRESpuro3 Vector (631619; Takara) using Nhe I and Xho I. All constructs were confirmed by direct sequencing. UHRF1 was transiently overexpressed by transfection of the resulting plasmid vectors into TE-6 or KYSE30 cells, which exhibit low UHRF1 expression, using Lipofectamine 3000 (Invitrogen). Cells subjected to mock transfection were used as a control. cDNA fragments encoding amino acids 1 through 112 of human *MBD1*, corresponding to the MBD and nls coding regions, were cloned into the *pEGFP-C1* vector (Clontech, Mountain View, CA) as previously described by Fujita et al. [28].

### 5AZA treatment

Cells were seeded in a 100-mm dish for 24 h. To demethylate methylated CpG sites, cells were continuously treated with 5AZA (Wako, Osaka, Japan; 100 nM-concentration) over the next 72 h. The medium was replaced every 24 h.

### Western blotting

Protein samples were subjected to sodium dodecyl sulfate-polyacrylamide gel electrophoresis, transferred to a nitrocellulose membrane, and exposed to primary antibodies. Signals were detected by incubation with secondary antibodies labeled using the ECL Detection System (GE Healthcare, Little Chalfont, UK). The primary antibodies, UHRF1 (1:1000 dilution, Abcam), GFP (1:1000 dilution, Cell Signaling) and β-actin (1:1000 dilution, Cell Signaling) were used in our study.

### Statistical methods

All statistical calculations were performed with JMP version 11.2 (SAS Institute Inc., Cary, NC, USA) and Excel for Mac 2011 (Microsoft, Redmond, WA, USA). For survival analysis, the Kaplan–Meier method was applied to evaluate the survival time distribution and the log-rank test was used for comparisons. We constructed a UHRF1-adjusted Cox proportional hazard model to calculate the hazard ration according to the LINE-1 methylation status. A multivariate logistic regression model initially included age (continuous), sex, tumor location (upper vs. lower), tumor depth (mucosal or submucosal layer vs. muscular or deeper layer), lymph node metastasis (negative vs. positive), lymphatic invasion (negative vs. positive), vascular invasion (negative vs. positive), histopathological types (well to moderate vs. poor), LINE-1 methylation level (hypermathylation vs. hypomethylation), and UHRF1 expression (negative vs. positive). In the univariate analysis, all variables with *P*-value < 0.10 were applied to the multivariate analysis. We considered statistical differences as significant at *P* < 0.05.

## SUPPLEMENTARY MATERIALS FIGURES



## Abbreviations

CSS: cancer-specific survival
ESCC: esophageal squamous cell carcinoma
HRs: hazard ratios
LINE- 1: long interspersed nucleotide element-1
OS: overall survival
PCR: polymerase chain reaction
qRT-PCR: quantitative reverse transcription PCR
siNC: normal control siRNA
siUHRF1: small interfering RNA for UHRF1
UHRF1: ubiquitin-like with PHD and RING finger domain 1.
