# Supplementary material for: UHRF1 regulates global DNA hypomethylation and is associated with poor prognosis in esophageal squamous cell carcinoma
Source: Oncotarget. 2016 Aug 5;7(36):57821–31. doi: 10.18632/oncotarget.11067 (PMC5295392; doi:10.18632/oncotarget.11067)
Supplement: Supplementary file 1 [file oncotarget-07-57821-s001.pdf]

UHRF1 regulates global DNA hypomethylation and is associated with poor prognosis in esophageal squamous cell carcinoma

Supplementary Materials

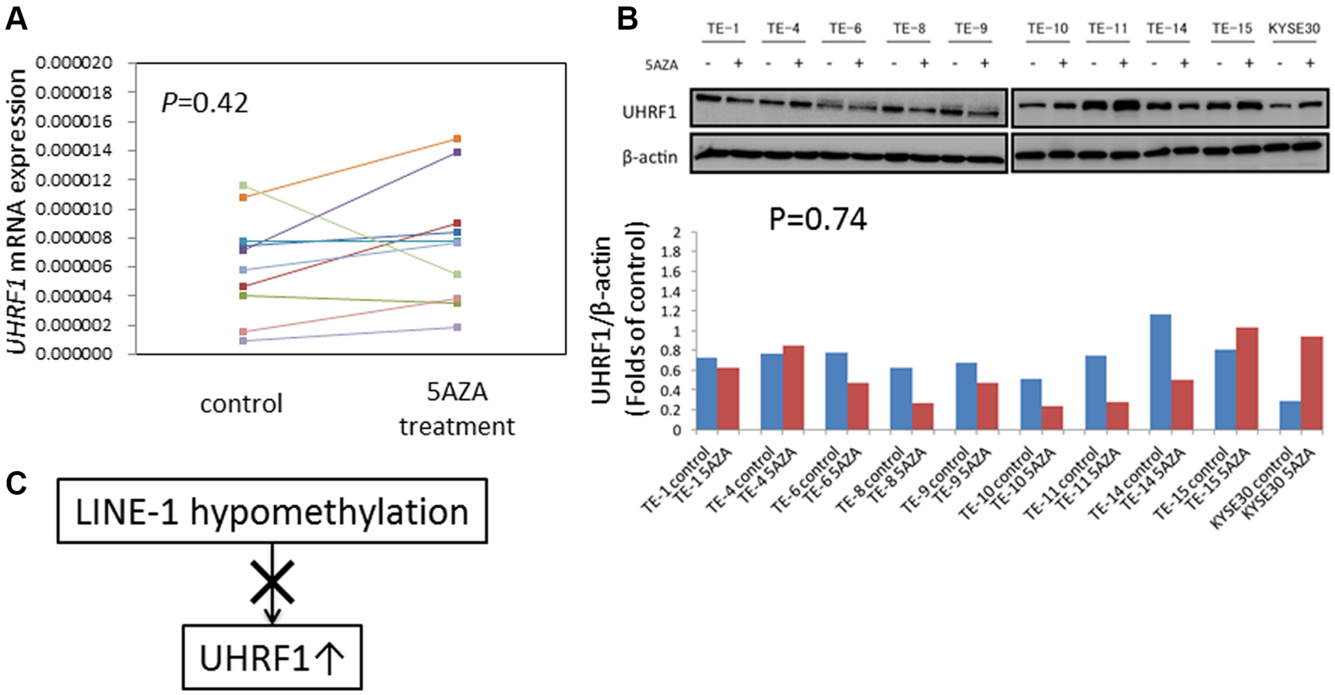

Supplementary Figure S1: Changes of expression of UHRF1 level in ESCC cell lines treated with 5AZA. (**A**) *UHRF1* mRNA expression level did not change after 5AZA treatment ($P = 0.42$). (**B**) UHRF1 protein expression level also did not change after 5AZA treatment ($P = 0.74$). (**C**) LINE-1 hypomethylation does not affect UHRF1 expression.

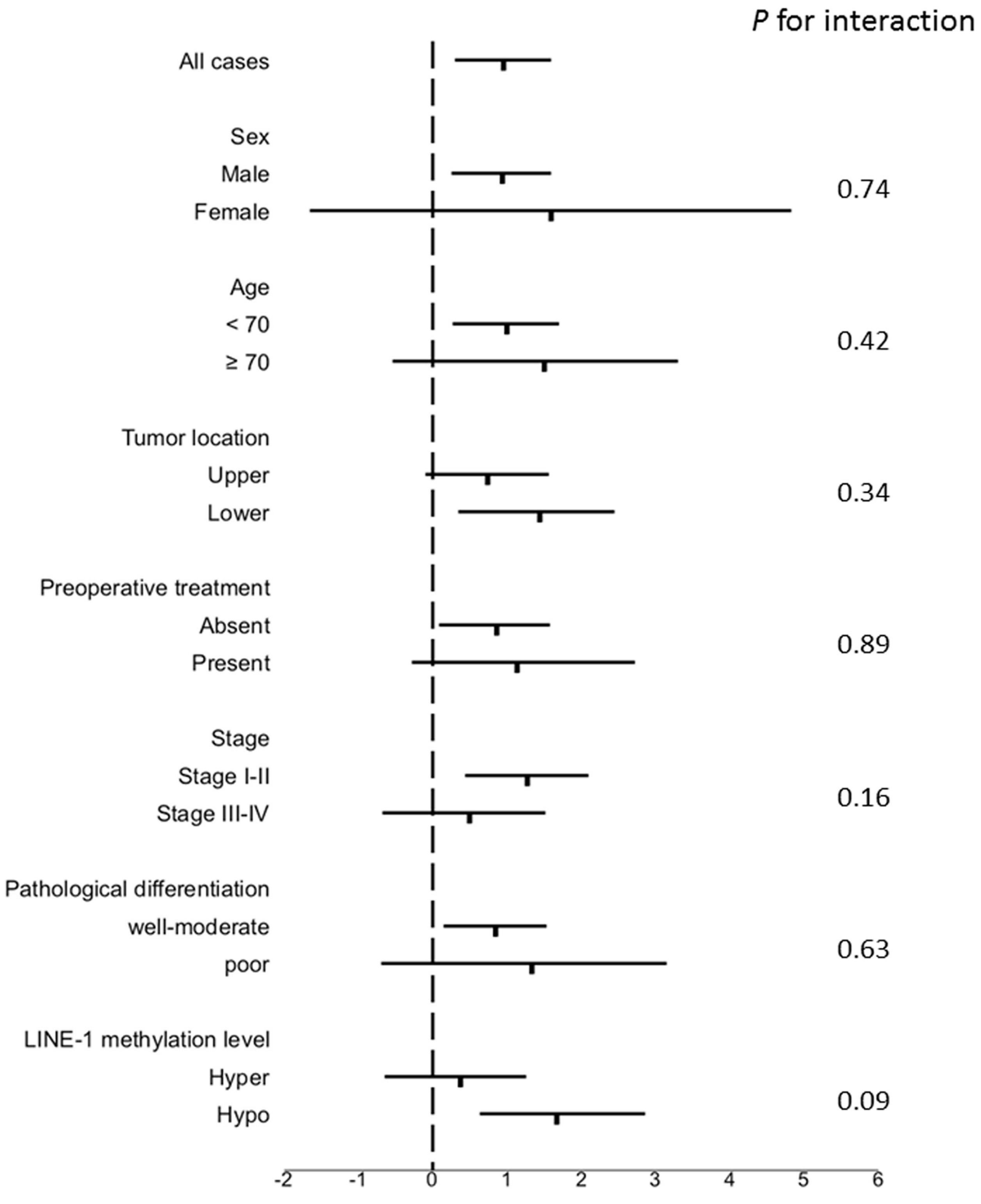

Supplementary Figure S2: UHRF1 expression and overall survival in various strata. Log_e (HRs) plots showing the 95% CI for the overall survival rate related to UHRF1 overexpression.
